# New Weapons to Fight against *Staphylococcus aureus* Skin Infections

**DOI:** 10.3390/antibiotics12101477

**Published:** 2023-09-22

**Authors:** Eliana M. Cela, Dolores Urquiza, Marisa I. Gómez, Cintia D. Gonzalez

**Affiliations:** 1 Consejo Nacional de Investigaciones Científicas y Técnicas (CONICET), Buenos Aires C1425FQB, Argentina; elianamcela@gmail.com (E.M.C.); urquiza.dolores@maimonides.edu (D.U.); gomez.marisa@maimonides.edu (M.I.G.); 2 Cátedra de Inmunología, Facultad de Farmacia y Bioquímica, Universidad de Buenos Aires, Buenos Aires C1113AAD, Argentina; 3 Centro de Estudios Biomédicos, Básicos, Aplicados y Desarrollo (CEBBAD), Departamento de Investigaciones Biomédicas y Biotecnológicas, Universidad Maimónides, Buenos Aires C1405BCK, Argentina; 4 Departamento de Microbiología, Parasitología e Inmunología, Facultad de Medicina, Universidad de Buenos Aires, Buenos Aires C1121ABG, Argentina

**Keywords:** *Staphylococcus aureus*, skin infections, antibiotic resistance, drug delivery, drug bioavailability, experimental therapies

## Abstract

The treatment of *Staphylococcus aureus* skin and soft tissue infections faces several challenges, such as the increased incidence of antibiotic-resistant strains and the fact that the antibiotics available to treat methicillin-resistant *S. aureus* present low bioavailability, are not easily metabolized, and cause severe secondary effects. Moreover, besides the susceptibility pattern of the *S. aureus* isolates detected in vitro, during patient treatment, the antibiotics may never encounter the bacteria because *S. aureus* hides within biofilms or inside eukaryotic cells. In addition, vascular compromise as well as other comorbidities of the patient may impede proper arrival to the skin when the antibiotic is given parenterally. In this manuscript, we revise some of the more promising strategies to improve antibiotic sensitivity, bioavailability, and delivery, including the combination of antibiotics with bactericidal nanomaterials, chemical inhibitors, antisense oligonucleotides, and lytic enzymes, among others. In addition, alternative non-antibiotic-based experimental therapies, including the delivery of antimicrobial peptides, bioactive glass nanoparticles or nanocrystalline cellulose, phototherapies, and hyperthermia, are also reviewed.

## 1. Introduction

*Staphylococcus aureus* is the primary cause of skin and soft tissue infections (SSTIs) worldwide [1]. Current treatment of *S. aureus* SSTIs is a challenge due to the emergence of multi-drug resistance strains, particularly methicillin-resistant *S. aureus* (MRSA). The majority of MRSA isolated from SSTIs are community circulating strains and are associated with complicated infections [2,3,4]. Antibiotic resistance is not the only problem that clinicians have to face in treating *S. aureus* SSTIs. Bioavailability is a key factor for antibiotic efficacy, and this might be seriously compromised by hydrophobicity, reduced half-life, and/or deficient tissue penetration [5]. To overcome these problems, parenteral administration of the antibiotics or the use of higher doses are often required, with the undesirable consequences of increasing antibiotic side effects and toxicity. In addition to antibiotic resistance and bioavailability, another important issue is the fact that antibiotics may never reach the bacteria because *S. aureus* hides within the host cells or protects itself by living in bacterial communities forming biofilms. To add to the problematic of staphylococcal SSTIs, in complicated infected ulcers, particularly in patients with comorbidities such as diabetes mellitus or vascular diseases, parenteral administration of the antibiotics is not efficient because the antibiotic is not able to reach the local niche where the bacteria replicates due to the vascularly compromised condition of the patient [4].

The present manuscript aims to revise novel strategies that are currently under study in order to develop SSTI treatments leading to bacterial elimination and wound healing. Here we describe different strategies that were designed to increase penicillin and methicillin sensitivity, improve the bioavailability and stability of antibiotics, and/or improve skin penetration during topical treatments. Non-antibiotic-based strategies including antimicrobial peptides, phototherapies, and nanotechnologies involving magnetic hyperthermia or bioactive metallic particles are also reviewed. Numerous studies have documented the antibacterial action of essential oils (EOs) against *S. aureus.* However, the potential of using EOs is not reviewed in this work due to the elevated toxicity of these compounds against human cells [6,7,8], which limits their clinical applications. Additionally, after exposure to sub-inhibitory doses of several EOs, the induction of antibiotic resistance was documented [9]. Bacteriophage-based therapies were recently reviewed by Plumet et al. and, for that reason, were excluded from this manuscript [10].

## 2. Novel Strategies to Increase Sensitivity to Currently Available Antibiotics as well as Their Bioavailability, Stability, and Tissue Penetration

### 2.1. Restoring Sensitivity to β-Lactam Antibiotics

**Figure 1:**
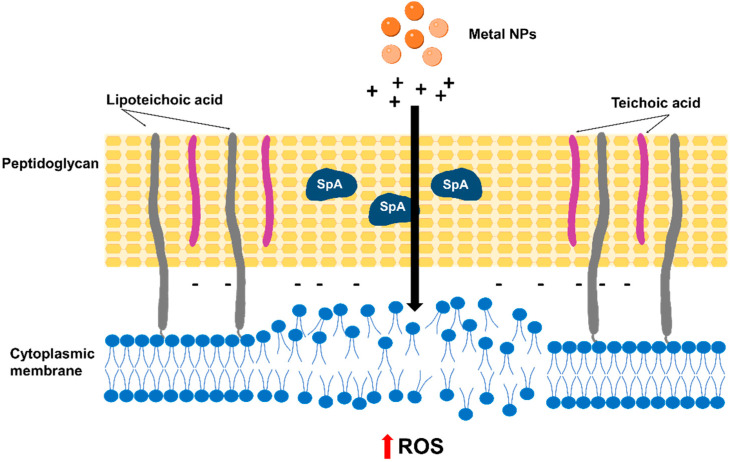
Action of nanoparticles on the bacterial membrane. Metal nanoparticles (NPs) release positively charged ions that alter membrane function, which leads to increased intracellular ROS levels and eventually cause bacterial destruction. NPs can be conjugated to different drugs (including antibiotics) to facilitate their delivery and may have a synergistic effect on bacterial killing.

Metal nanoparticles (NPs) have been recently evaluated to be used in combination with β-lactam antibiotics or as carriers to improve their efficacy. The combination of cefotaxime (Ctx) with Bi_2_Te_3_ NPs showed a significant synergistic antibacterial activity against MRSA, reducing the minimal inhibitory concentration (MIC) of Ctx from 256 to 32 µg/mL [11]. Bi_2_Te_3_ NPs partially inhibit β-lactamase production and alter membrane function, which lead to increased intracellular reactive oxygen species (ROS) levels and improved Ctx internalization, eventually causing bacterial destruction [11] (Figure 1). Gold NPs were used as nanocarriers for ampicillin (Amp). Although gold NPs are inert, Amp-Au NPs leave the β-lactam ring free to interact with the bacterium and have been shown to improve the bacterial sensitivity to the antibiotic in vitro [12]. Moreover, Amp-Au NPs accumulate on the bacterial surface and trigger the formation of pores that facilitate antibiotic entry while protecting ampicillin from other bacterial defenses such as multidrug exporters/multidrug efflux pumps [12]. 

Another strategy that has been proposed to overcome resistance is the blocking of β-lactamases. In this regard, Thomas et al. used pyrimidine-2-amines (P2As) as a new inhibitor that, through an unknown regulator, prevents *blaZ* expression and therefore suppresses β-lactamase activity in vitro [13]. The presence of P2As enhanced penicillin G sensitivity and reduced the MIC of Amp against MRSA USA300 from 256 μg/mL to 64 μg/mL [13].This approach has the advantage of not employing direct enzyme inhibitors such as sulbactam and clavulanate that can contribute to the development of resistance [14].

**Figure 2:**
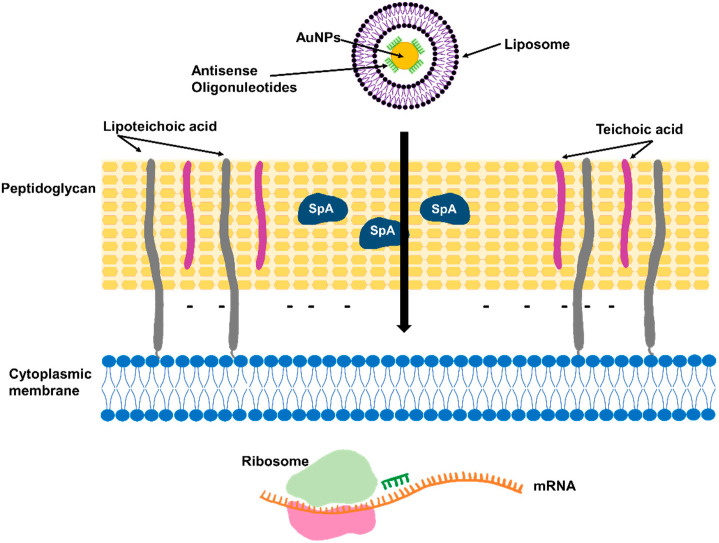
Antisense technologies to restore sensitivity to β-lactam antibiotics. Antisense oligonucleotides coupled to gold nanoparticles (AuNPs) delivered using liposomal nanocarriers can block gene expression of *mecA* or *mecR1* to restore sensitivity to methicillin.

Methicillin resistance requires the SSC*mec* (staphylococcal cassette chromosome *mec*) mobile genetic element that harbors the *mecA* gene. This gene encodes PDP2a, a receptor with low affinity for β-lactam antibiotics, which confers resistance to penicillins, cephalosporins, and carbapenems [15]. Antisense technology has been used to block PDP2a expression, and, as a consequence, the sensitivity to β-lactam antibiotics was improved [16,17] (Figure 2). Meng et al. used a liposomal formulation to administer phosphorothioate oligodeoxynucleotides (PS-ODNs) that block *mecR1*, leading to a decrease in the expression of both *mecR1* and *mecA*; as a result, a reduction in the MICs of oxacillin, floxacillin, cefoxitin, cephalothin, and cefoperazone against MRSA strains was observed. Increased survival of mice treated with PS-ODNs and oxacillin was observed in a sepsis infection model [16]. Recently, multi-layer coated gold nanoparticles (MLGNPs) were used to deliver antisense oligonucleotides (ASOs) targeting the *mecA* gene [17]. Using a suspension of human cells and bacteria, it was shown using A549 human lung cancer and HaCaT human epidermal keratinocyte cells that these MLGNP-ASOs were preferentially internalized in bacteria over eukaryotic cells, with very little antibacterial action or cytotoxicity. Treatment of MRSA with MLGNP-ASOs targeting *mecA* reduced the expression of the gene by 74%, indicating that the resistance gene was effectively silenced [17]. Additionally, in oxacillin agar plates, the treatment reduced bacterial growth in a target-specific and concentration-dependent manner, suggesting that MLGNP-ASOs were able to restore antibiotic sensitivity [17].

Recently, Sharaf et al. showed that the combination of penicillin with the active saponin fraction isolated from the *Zygophyllum album* plant (ZA-S) enhanced its activity against MRSA [18]. The MIC against MRSA was 250 µg/mL and 1250 µg/mL for penicillin and ZA-S, respectively, when evaluated alone, whereas the combined treatment had an MIC of 62.5 µg/mL and 312.5 µg/mL for penicillin and ZA-S, respectively [18]. The combination of penicillin with ZA-S showed no cytotoxic effect in Vero, MRC-5, and HBF4 cell lines using the MTT cytotoxicity assay. The synergistic effect can be explained by the detergent-like properties of saponins, which might increase the permeability of bacterial cell membranes and, therefore, antibiotic influx through the bacterial envelope, increasing MRSA sensitivity to penicillin [18].

### 2.2. Increasing Bioavailability of Hydrophobic Antibiotics: Macrolide and Fluoroquinolones

**Figure 3:**
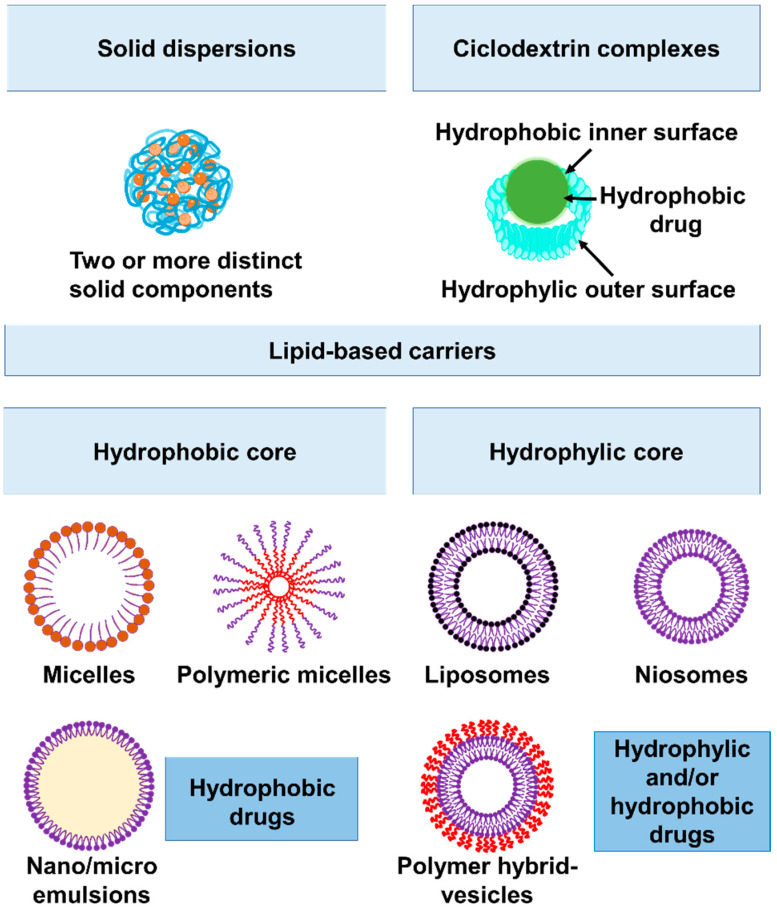
Nanocarriers used to improve drug delivery. Solid dispersions: these are formed by two or more solid components, generally a hydrophilic polymeric matrix (such as PVP or PEG) and a hydrophobic drug. Cyclodextrins: the cylindrical shape allows the drug to be kept within the hydrophobic interior, while the outer surface is hydrophilic and soluble in aqueous solutions. Lipid-based carriers: Micelles, polymeric micelles, and nano- or microemulsions are composed of a monolayer (one or more surfactants) and a hydrophobic core and serve to carry hydrophobic drugs. Vesicles have a hydrophilic core and, according to their composition, can be classified as liposomes (phospholipids), niosomes (non-ionic surfactants with or without cholesterol) or polymer-hybrid vesicles (liposomes or niosomes coated with polymers) and serve to carry hydrophobic or hydrophilic drugs.

Solubility is critical for parenteral formulations in order to achieve a concentration of the drug in circulation that is required for a pharmacological response while minimizing undesirable side effects [19,20]. Poor solubility of antibiotics in aqueous solutions is a frequent reason for insufficient drug bioavailability. Several delivery methods improve the physiologically active compound solubility and bioavailability and, thus, reduce their toxic effects as well as increase drug stability while in storage. Among the plethora of nanotechnologies currently available to improve drug delivery, solid dispersions (SDs) consist of two or more different solid components, usually a hydrophilic polymeric matrix and a hydrophobic drug [21] (Figure 3). Other strategies include lipid-based formulations (such as liposomes, niosomes, polymersomes), nanocrystals, and complex formations with regular or modified cyclodextrins [22] (Figure 3). Several of these formulations are currently under study to improve the efficacy of antibiotics with poor solubility in aqueous solutions, such as macrolides and fluoroquinolones, against *S. aureus*. Naskar et al. produced a nanocomposite that consisted of Ag^+^ and polyvinylpyrrolidone (PVP-40000) uniformly distributed along a ZnO nanostructure [23]. The nanocomposite, named AZO, was used as a nanocarrier for erythromycin (Em). Scanning electron microscopy (SEM) revealed significant membrane damage caused by the synergistic killing effect of AZO-Em on *S. aureus* [23]. Electrostatic interactions between the nanocomposite Zn^2+^/Ag^+^ ions and the negatively charged bacterial membrane results in increased membrane damage, which allows AZO-Em to enter the cell and favor protein synthesis inhibition by Em. Additionally, the membrane damage disrupts bacterial cell function and might result in intracellular material leakage that ultimately leads to cell lysis (reviewed by Slavin et al. [24]). Compared with antibiotics alone, the AZO-Em nanocomposite had a lower risk of drug resistance, as demonstrated by performing eight passages using sub-inhibitory concentrations. In addition, the synthesized nanocomposite showed high biocompatibility with HEK 293T human embryonic kidney cells. Altogether, the in vitro analyses suggest that the AZO-Em nanocomposite can be employed as an effective antibacterial agent. Upcoming in vivo studies will determine whether it is feasible to use this formulation to treat *S. aureus* infections.

Another type of Ag-based nanoparticle has been elaborated to function as a ciprofloxacin (Cip) nanocarrier by Ibraheem et al. [25]. In this case, the NPs were synthetized by the reduction of AgNO_3_ to obtain metal Ag^0^ NPs that were then attached to the synthetic polymer polyethylene glycol (PEG). PEG enhanced the biocompatibility of the nanoparticles and worked as a linker between the AgNP surface and Cip [26]. The disk-diffusion susceptibility test was used to determine the inhibitory activity of AgNPs and their conjugates [25]. Cip-AgNPs increased antibiotic internalization as measured by the inhibition of *S. aureus* growth with an inhibition zone of 35.33 ± 1.52 mm, compared with inhibition zones of 29.33 ± 1.80 mm and 16.00 ± 2.12 mm when using AgNPs alone or Cip alone, respectively. PEG-Cip-AgNPs demonstrated better antibacterial activity than the other formulations (inhibitory zone = 39 mm) [25], suggesting that the addition of PEG considerably enhanced *S. aureus* sensitivity to Cip. Improved antibacterial activity of PEG-Cip-AgNPs resulted from the slow release of the loaded Cip molecules from the nanoconjugate [27]. The antibacterial activity of the synthesized AgNPs is highly dependent on size and shape due to the vertexes and the numerous sharp edges of the nanoprisms that may facilitate the penetration of the AgNP nanoformulation across and into the cell walls [28].

Mendes et al. employed cyclodextrin-based nanoparticles to boost oral absorption of the fluoroquinolone norfloxacin (Nfx) [29]. CD is a nanoparticle that consists of a cyclic oligosaccharide containing seven glucose units linked by α-(1,4) bonds. These particles possess a hydrophilic exterior surface and a hydrophobic interior cavity that allows the loading of a range of hydrophobic molecules into inclusion complexes [30]. The efficacy of treatment with CD-Nfx compared with free Nfx has been evaluated in a rat sepsis model of cecal ligation and puncture (CLP). The bacterial load in the kidney 24 h after CLP was significantly lower in the CD-Nfx-treated group than that in the Nfx-treated and the untreated groups. The improved bacterial clearance may be due to an increased adherence to the intestinal mucosa and permeability of the nanoconjugates [29].

CD-based nanoparticles were also implemented as moxifloxacin (Mox) carriers [31]. Despite the fact that the MIC against MRSA did not differ between CD-Mox and free Mox, CD complexation was able to increase the solubility of the drug and prevented oxidation and light-induced degradation [30]. In this context, in vivo experiments using CD-Mox will determine whether there is an improvement in the pharmacokinetics of the antibiotic and amelioration of secondary effects in comparison with the free form.

Recently, liposomal formulations have been evaluated in order to enhance the delivery of Nfx. Tanase et al. proposed the use of polymeric mixed micelles made from the nonionic surfactants Pluronic F127 and Cremophor EL [32]. Although the formulation slightly improved the antibacterial activity of Nfx against *E. coli*, no reduction in the MIC against *S. aureus* was observed, indicating that modifications to the composition of the formulation are required.

Akbar et al. recently employed metronidazole-based niosomal vesicles (MNVs) as Mox nanocarriers [33]. Mox-loaded MNVs showed dramatically improved bactericidal activity against MRSA and other multidrug-resistant bacteria compared with the free drug. Additionally, these drug-loaded nanocarriers presented good biocompatibility with human cells (as determined by cytotoxicity assays using Hela cells) [33]. 

### 2.3. Increasing Bacterial Uptake of Antimicrobials: The Trojan-Horse-like Strategy

**Figure 4:**
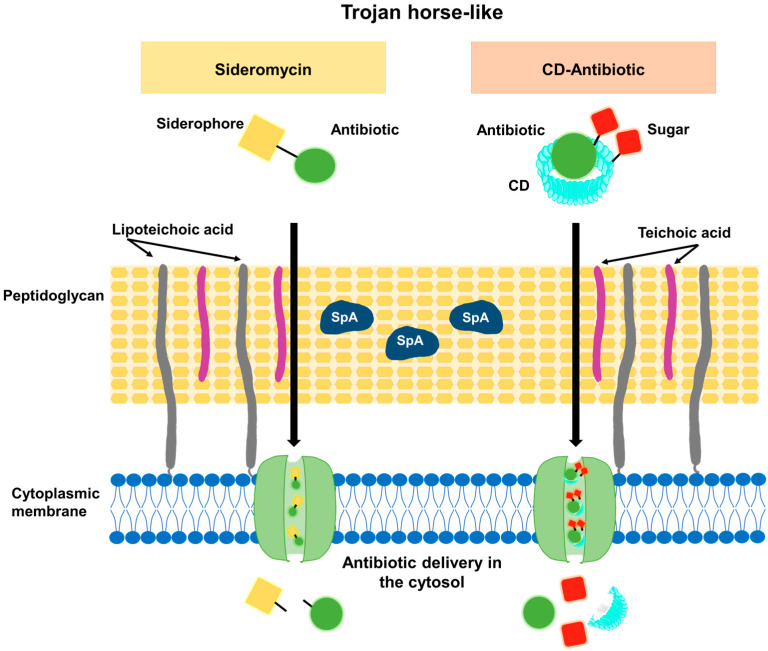
Trojan-horse-like strategy. Siderophores or sugar-crafted cyclodextrins can facilitate antibiotic delivery through the uptake by their specific siderophore or sugar transporters in the cytoplasmic membrane and subsequent release of the antibiotic in the bacterial cytoplasm.

For those antibiotics with a cytosolic target, efficacy can be increased by enhancing the bacterial uptake of the drug. In this regard, it is feasible to take advantage of nutrient transporters and to use them as an acquisition route. Siderophores are iron chelators secreted by the bacteria to increase the uptake of iron, an essential and scarce nutrient in the infection microenvironment [34]. Certain bacterial species produce siderophores conjugated to antibiotics. These siderophores capture iron, and the entire conjugate is internalized by the bacteria. After internalization, the antibiotic is liberated in the cytosol, killing the bacteria unless an efflux mechanism provides resistance (Figure 4). These molecules are known as sideroantibiotics or sideromycins and play a natural role in the eradication of other competitive microorganisms [35]. Among sideromycins, salmycins are formed by linear siderophores and show selective antimicrobial activity against Gram-positive pathogens, including *S. aureus*. However, they exhibit weak action in vivo, likely due to extracellular hydrolysis of the labile ester bond [35].

Wencewicz et al. have produced synthetical conjugates inspired by salmycin B, a sideromycin consisting of the linear siderophore hydroxamate conjugated to a glycopeptide antibiotic. Using β-lactam antibiotics or fluoroquinolones as a replacement for the glycopeptide antibiotic, several compounds were obtained. An important limitation of the use of sideroantibiotics as a tool to increase antibiotic uptake by the bacteria is that the concentration of iron in the environment may determine the activity of the siderophore. Mono-, bis-, and trihydroxamate sideroantibiotics were studied, and their activity, measured by their MIC, was compared with that of free antibiotics in the presence of different iron concentrations. Among these compounds, trihydroxamate conjugated with the fluoroquinolone Cip showed an efficacy equivalent to that of the free antibiotic, regardless of the iron concentration [36]. Future studies will determine the in vivo safety of these synthetic sideromycins and their potential to be used for the treatment of *S. aureus* infections.

Albomycins are another type of sideromycin with broad-spectrum antibacterial activity in vitro and in vivo against both Gram-positive and Gram-negative bacteria [37,38]. They contain a tri-δ-N-hydroxy-L-ornithine peptide siderophore joined through an amide bond to a thioribosyl pyrimidine inhibitor of the seryl-tRNA synthetase [39]. For *Streptococcus pneumoniae* strains, the MIC of albomycins ranges from 4 to 62 nM [38,40], and large-scale preclinical and clinical studies conducted at the beginning of the 1950s showed the albomycins’ exceptional safety and efficacy in treating meningitis and lung infections caused by penicillin-resistant pneumococci [41]. Recent improvements in complete chemical synthesis have made it possible to manufacture albomycin and its analogues on a large scale. Lin et al. synthesized albomycins δ1, δ2, and δ3 and evaluated their antibacterial activity against *S. aureus*. Albomycin δ2 showed efficacy against the MRSA USA 300 strain NRS38441, with an MIC of 0.125 µg/mL, which is 16 times more potent than Cip [40]. SB-217452, the seryl-linked nucleoside moiety released from albomycin δ2 upon the cleavage of the siderophore region, was shown to inhibit *S. aureus* seryl-tRNA synthetase (SerRS) at a nanomolar concentration [39,42]. Additional research is needed to determine the safety of albomycin δ2 in vivo and its effectiveness for the treatment of *S. aureus* infections [40]. 

Another Trojan-horse-like strategy was developed by Li et al. [43]. They used sugar-grafted β-cyclodextrins (CD) as antibiotic nanocarriers (Figure 4). CD was grafted with either D-mannose or D-glucose (CD-MAN or CD-GLU carriers, respectively). Mannose and glucose are both carbon sources for bacteria and, therefore, they can easily be internalized through sugar transporters in the cell membrane [44], making the bacterium ingest the antibiotic simultaneously (Figure 4). The authors used this carrier to transport the hydrophobic antibiotic Em. In contrast to the free antibiotic, which has limited solubility in aqueous solutions (≤1 mg/mL), the CD-MAN-Em and CD-GLU-Em complexes present high solubility. The antibacterial efficacy of free Em, CD-MAN-Em, CD-GLU-Em, and CD-Em complexes has been evaluated. The MIC against the MRSA strain susceptible to Em (MRSA DM23605) [45] was reduced by a factor of 3.3 using CD-MAN-Em compared with free Em. For the highly Em-resistant strains, MRSA DR9369 and ATCC BAA-44, a reduction in the MIC from 1024 mg/L to 4.8 mg/L and 76.8 mg/L, respectively, was observed using the CD-MAN-Em in comparison with free Em. The Gram-negative *P. aeruginosa* (PAO1) and its efflux-deficient derivative strain were used to demonstrate that the CD-MAN carrier is able to potentiate the activity of the loaded antibiotic, even in efflux-proficient bacterial strains [43]. Similar results were obtained with CD-GLU-Em. CD-MAN nanocarriers not only enhanced Em internalization and effectiveness but also decreased the risk of acquiring resistance [43]. To evaluate the acquisition of resistance in *S. aureus*, the ATCC 25923 strain was incubated with sub-inhibitory concentrations of CD-MAN-Em, CD-Em, or free Em (0.25 times the MIC of free Em) for 16 days, and then suspensions were plated in TSB agar containing two times the MIC of free Em. One in approximately 2 × 10^4^ bacteria developed resistance to free Em and CD-Em, whereas colonies resistant to CD-MAN-Em were not found. Moreover, CD-MAN-Em does not present significant cytotoxicity in mammalian cells, as tested in RAW 264.7 macrophages, human NCM460 colonic epithelial cells, and HEK 293T cells, using a concentration that corresponds to 16 times the MIC of Em for *P. aeruginosa* PAO1 or 3 times the MIC of CD-MAN-Em for *S. aureus* ATCC BAA-44 [43]. 

### 2.4. Improving Local Delivery of Antibiotics during Skin Infections

Nanotechnologies provide a wide spectrum of possibilities for the delivery of antimicrobial molecules. Liposomal nanoparticles, microemulsions, and niosomal vesicles (Figure 3) are promising vehicles since they have properties that are required for topical treatments, such as promoting or facilitating skin penetration, increasing drug stability, and providing a slow and extended release.

Vancomycin (Vm) is one of the limited options of primary antibiotics used to treat MRSA. This hydrophilic glycopeptide, although soluble, is not only markedly unstable but also possesses low oral bioavailability and considerably low tissue penetration. In particular, its skin and soft tissue penetration ranges from 30% in the skin of individuals without comorbidities to 10% in the skin of diabetic patients [46,47,48,49]. In this regard, the development of nanoformulations that improve Vm stability and tissue penetration is critical for the treatment of MRSA SSTIs. Novel lipid–based nanocarriers are under study for the delivery of Vm. The oleylamine-based lipid (OLA) has been used in conjugates with hyaluronic acid to develop polymersomes (PS6) [50] or to formulate chitosan-based pH-responsive lipid–polymer hybrid nanovesicles (OLA-LPHNVs1) [51]; both formulations were loaded with vancomycin. Vm-loaded polymersomes showed sustained release of the drug for 72 h and its bactericidal activity was four times the activity of free Vm [50]. The OLA-LPHNVs1-Vm nanovesicles represented a greater improvement as antibacterial agents, with MICs against MRSA corresponding to 2.39 µg/mL at pH 7.4 and 0.59 µg/mL at pH 6.0, in contrast with free Vm, which had an MIC of 31.25 µg/mL at both pH conditions [51]. A significant decrease in the biomass of biofilms treated with OLA-LPHNVs1-Vm was observed compared with Vm-treated and untreated biofilms [51]. Furthermore, during in vivo experiments using a mouse model of skin infection, subcutaneous treatment with OLA-LPHNVs1-Vm induced a 95-fold increase in MRSA clearance at 48 h post-treatment compared with the group treated with free Vm [51]. The enhancement in the antimicrobial activity of OLA-LPHNVs1-Vm in comparison with free Vm could be due to the increase in the binding of OLA-LPHNVs1-Vm to the negatively charged bacterial membrane, as reported for other positively charged chitosan-based formulations [52].

The effectiveness of nanocarriers and their ability to cross the stratum corneum needs to be determined, since the skin barrier presents low permeability unless proper exogenous physical stimuli are provided [53]. In order to enhance tissue penetration and the skin accumulation of Vm, different platforms are under study. Argenziano et al. loaded Vm onto nanobubbles (NBs) composed of a perfluoropentane (PFP) core and a dextran sulfate shell [54]. Incorporation of Vm into the nanobubbles significantly improved its stability and its antibacterial efficacy in comparison with the free drug. Additionally, Vm-PFP-NBs antibacterial effects appeared as early as 2 h of incubation, whereas free Vm required 3 to 4 h to act. Vm-PFP-NBs were found to adhere to the bacterial surface of MRSA, whereas free Vm was avidly internalized. The ability of ultrasound (US) to promote the permeation of Vm through the skin was assayed by employing a purposely modified Franz cell constituted by a donor and a recipient chamber separated by a porcine skin layer. The administration of US (t = 10 min; f = 2.5 MHz; P = 5 W) strongly induced Vm-PFP-NBs to deliver the antibiotic from the donor chamber throughout the pig skin membrane into the recipient chamber over 6 h. Furthermore, the drug accumulated in the skin after US treatment reached 158 µg/cm^2^ after 6 h. The combination of NBs and US enhanced Vm permeation through pig skin and promoted drug accumulation in the skin. Based on these results, Vm topical administration through proper NB formulations might be a promising strategy for the local treatment of MRSA skin infections.

Another platform to enhance tissue penetration has been proposed by Dhanalakshmi et al. and consists of chitosan nanoparticles (CNPs) coated with lecithin (CLNPs) [55], which have been used to deliver tigecycline (Tig). Lecithin coating enhanced the stability of chitosan nanoparticles (CLNPs) loaded with Tig at pH 8.0, enabling a gradual and sustained Tig release rather than a burst [52,56]. Skin penetration studies showed that Tig released from Tig-CLNPs penetrated the skin more deeply than that released from Tig-CLNPs or free Tig [55]. The lipid-coated nanoparticles adhere to the surface and penetrate through the lipid covering of skin due to the occlusive effect [57]. The lipid coating of Tig-CLNPs also enhanced the subsequent release of the drug from the nanoparticles. Altogether, these results indicate that Tig-CLNPs can effectively deliver Tig through the skin and soft tissue [55], which encourages further studies to determine whether they could be used in the treatment of complicated SSTIs.

Microemulsions (MEs) are known to improve skin retention due to their interaction with skin lipids [58], which makes them a promising option for local drug delivery. Abruzzo et al. produced MEs composed of vitamin E acetate, Labrasol^®^, and Transcutol^®^ P as azithromycin (Azt) carriers [59]. Formulations M1, M2, and M3 loaded with Azt were obtained using different percentages of each compound. Skin penetration/retention studies through porcine skin as the interface between a donor chamber and a receptor chamber showed higher accumulation in the skin with a 46.69%, 59.58%, and 36.65% for M1, M2, and M3 respectively, whereas the amount of free Azt retained inside the skin was 22.04%. The lower retention of free Azt was caused by an increased accumulation of Azt in the receptor chamber (64.13% compared with less than 26% for the MEs). These findings clearly indicate that microemulsions could reduce undesired Azt penetration through the skin, which is fundamental for the treatment of topical skin infections while also considering the need for lowering systemic Azt absorption.

## 3. Novel Non-Antibiotic-Based Strategies for the Local Treatment of SSTIs

### 3.1. Local Delivery of Antimicrobial Peptides

Antimicrobial peptides (AMPs) are a promising alternative for the treatment of bacterial infections [60]. AMPs are usually broad-spectrum positively charged bactericidal molecules that bind selectively to negatively charged microbial membranes, and, through their hydrophobic portions, interact with membrane lipids, compromising membrane stability, forming pores, and, ultimately, causing bacterial death (a detailed review of AMPs and their mechanism of action has been conducted by Huan et al. [61]). LL-37 is a human AMP with a strong bactericidal effect against *S. aureus.* This AMP is not only effective against planktonic bacteria but also prevents biofilm formation and disaggregates mature *S. aureus* biofilm structures [62]. Sadeghi et al. used niosomes (Figure 4) (composed of surfactants, cholesterol, and dicetylphosphate at a ratio of 47:47:6) to encapsulate and deliver LL-37, alone or in combination with lysostaphin [63]. The enzymatic action of lysostaphin on the bacterial cell wall increases *S. aureus* susceptibility to AMPs, and a synergistic effect with specific AMPs has been reported [64,65]. The antibacterial activity against *S. aureus* over time was determined for the niosome-encapsulated and free forms of LL-37, lysostaphin, and LL-37/lysostaphin. The free drugs were consumed early during the bacterial culture and then the *S. aureus* population began to grow, whereas encapsulated single formulations significantly reduced *S. aureus* growth during the 72 h of incubation [63]. Furthermore, the inhibitory effect of lysostaphin/LL-37-encapsulated dual formulations was greater than that of encapsulated lysostaphin or encapsulated LL-37, implying that encapsulated lysostaphin/LL-37 acts synergistically as has been observed with the free forms of the drugs [63]. Niosomal formulations were more effective than the free forms, probably because niosomes can interact with the bacterial membrane and release elevated levels of the drugs close to the bacterial surface [66]. Therefore, lysostaphin/LL-37-encapsulated niosomes may provide extended antibacterial action at a lower dose.

The AMP melittin (Mel) is a cationic α-helical peptide of 26 amino acids and is the main component of honeybee venom [67,68]. Mel accumulates on the membrane and compromises its integrity, as demonstrated using FITC-labeled Mel and a propidium iodide (PI) uptake assay [69]. Mel increases PI uptake in a dose-dependent manner compared with untreated bacteria. In addition to membrane destruction, the peptide can also exert its antibacterial activity through interactions with intracellular targets such as DNA, RNA, and proteins. Mel has anti-MRSA activity in vitro and a therapeutic effect in MRSA-infected mice, using models of bacteremia and skin infections [70]. Mel antibacterial activity has also been confirmed in Vm-intermediate *S. aureus* (VISA) clinical isolates, with MICs in the range of 6.25–25 μg/mL [69]. Surprisingly, VISA present a greater susceptibility to Mel than the control strains ATCC 25923 and MRSA ATCC 43300 [69]. Mel, however, is a toxic and hemolytic peptide, and in order to be used therapeutically, an appropriate carrier is required [71]. Sangboonruang et al. evaluated niosome vesicles loaded with Mel (Mel-NISVs) for local delivery in the skin [69]. First, they tested the efficacy of the formulation in vitro. To that purpose, *S. aureus* ATCC 25923, MRSA ATCC 43300, and VISA isolate 87 were incubated with Mel-NISVs for 24 h; then, the bacterial growth was evaluated by CFU quantification. Mel-NISVs significantly inhibited bacterial growth in a dose-dependent manner, whereas empty NISVs showed no antibacterial effect. The Mel-NISVs’ potential to penetrate the dermis and epidermis has been evaluated in porcine ear skin explants using fluorescent labeling. Mel-NISVs reached the epidermis layer after 2 h of treatment and extended to the deeper layers (up to 820 μm of depth) at 4 h. These results confirm that the niosomal nanocarrier system has the ability to permeate the epidermis and dermis [69]. The effect of Mel-NISVs on bacterial skin infections was investigated using porcine ear skin models of undamaged and burned wound skin infection. Skin samples were infected with FITC-labeled *S. aureus* ATCC 25923 and subsequently treated with NR-labeled Mel-NISVs for 4 h. As expected, *S. aureus* colonized the undamaged skin surface and spread into deeper skin layers of the wound burn area. Treatment of the infected skin with Mel-loaded NISVs resulted in decreased amounts of FITC-labeled *S. aureus* in the deeper layers of both the undamaged and burned wound skin models. These findings strongly suggest that the use of Mel-loaded NISVs may be affective against *S. aureus* infection [69].

### 3.2. Local Administration or Production of Nitric Oxide

Nitric oxide (NO) is a strong antimicrobial agent that damages bacterial membranes, proteins, and DNA, resulting in bacterial cell death [72,73]. NO induces nitrosative and oxidative stress mediated by nitrogen oxide intermediates (RNOS) [74], involving multiple mechanisms of action that might be used to fight against drug-resistant bacteria, including MRSA [75]. Moreover, the development of bacterial resistance against NO is remote because it would require the accumulation of several mutations [76]. In addition to its antimicrobial properties, NO has emerged as an exciting option for the treatment of infected wounds due to its beneficial effects in modulating inflammation, promoting cell proliferation and tissue remodeling, which, in turn, favors wound healing [77,78,79,80,81]. However, NO has a short migration distance in solution [82], and for that reason, an appropriate delivery system with a sustained and slow release is required. Silica, gold, and liposome-based NPs loaded with different NO-donors have been produced, but an initial burst release limits their action [83,84,85,86,87,88]. Nurhasni et al. produced NPs using polyethylenimine (PEI) as the NO donor polymer. PEI/diazeniumdiolate (PEI/NONOate) was synthesized by making NO react with the secondary amine groups of PEI and be incorporated in the matrix of poly(lactic-co-glycolic acid) (PLGA) NPs (PLGA-PEI/NONOate) [89]. From PLGA-PEI/NONOate, NO was released in a sustained manner over a period of 6 days, whereas PEI/NONOate released NO with an initial burst (∼90%) in the first 2 h. The treatment of MRSA (USA300, 10^6^ CFU) with PLGA-PEI/NONOate at a dose of 10 mg/mL NPs with an NO release of 0.2 µmole/24 h resulted in complete bacterial elimination, in contrast with the treatment with PLGA-PEI NPs, which had no effect on bacterial viability. The positively charged surface of PLGA-PEI/NONOate facilitates the electrostatic binding of the NPs to the negatively charged bacterial surface, thereby increasing NO delivery and antibacterial activity [89]. PLGA-PEI/NONOate antibacterial activity and wound-healing properties were evaluated using a murine wound infection model. Mice were wounded, infected with *S. aureus* USA300 (6 × 10^8^ CFU), and 24 h later, they were topically treated with PLGA-PEI/NONOate or with PLGA-PEI. The PLGA-PEI/NONOate-treated group exhibited a significantly reduced wound area and clear re-epithelialization compared with the PLGA-PEI-treated or untreated groups, indicating that PLGA-PEI/NONOate resulted in favorable wound healing with accelerated wound size reduction [89]. Other NO donor nanoparticles evaluated, such as NO-releasing silica nanoparticles and NO-releasing metal nanoparticles, also enhanced antibacterial activity and promoted wound healing [89,90,91,92,93,94]. An important limitation of these formulations, however, is that they consist of non-biodegradable materials, such as polyethyleneimine and heavy metals, which can potentially be accumulated in the skin tissue [95,96,97]. 

Among the variety of different kinds of NO donors, S-nitrosoglutathione (GSNO) is one of the prominent choices for the development of wound dressings (such as hydrogels, films, and microparticles) to improve healing [98,99,100,101,102] because it is converted into glutathione (GSH), a powerful mammalian antioxidant, after releasing NO [103,104]. Nanoparticles containing GSNO have been produced [105,106,107,108]. However, due to its hydrophilicity, GSNO was lost during the nanoparticle production process and NO loading was compromised. This issue was solved by Lee et al. recently by conjugating the GSNO molecule with PLGA, which reduced GSNO loss and optimized NO loading in the NPs [109]. After conjugation, GSNO-PLGA was then used to produce GSNO nanoparticles (GPNPs) using an oil-in-water emulsion evaporation method [109]. The antibacterial activity of GPNPs against MRSA was significantly higher than that observed for GNSO using concentrations with a relative release of 2 mM NO [109]. Treatment of MRSA with GPNPs for 24 h at 37 °C reduced by more than 4 log10 the number of CFUs, whereas GSNO showed no effect on bacterial counts. The antibacterial effect of GPNPs was also verified using LIVE/DEAD^®^ BacLight™ and confocal microscopy [109]. The improved antibacterial activity of GPNPs compared with GSNO could be attributed to a more efficient delivery of NO to the bacteria, considering the short migration distance of NO in solution [82]. The potential therapeutic effect of GPNPs was evaluated using a murine wound infection model. The wound was treated with 20 mg of GPNPs or GPNO 48 h after the challenge with MRSA (6 × 10^5^ CFU per wound), and subsequent treatment was applied every 2 days. Ten days post-inoculation, the mean wound size in the GPNP-treated group had reduced by more than 70% in comparison with the initial size, whereas in the GSNO-treated or untreated groups, the wound size achieved approximately 50% of the initial size [109]. The histopathological analysis (hematoxylin and eosin staining, Masson’s trichrome staining, and Twort’s Gram staining) of wounds at 10 days post-inoculation indicated that the skin tissue from the GPNP-treated group was well differentiated (hair follicles, glands, and clear epidermis were observed), with abundant collagen deposition in the dermis region. In contrast, skin tissue from the GSNO-treated and untreated groups showed an inflammatory environment characterized by the presence of a large number of immune cells and granulocytes, decreased collagen deposition, and compromised recovery of the dermis and epidermis [109]. In addition, MRSA could easily be observed at the outer region of wound tissues taken from GSNO-treated and untreated mice, whereas bacteria were rarely observed in GPNP-treated and healthy mice [109]. Taken together, GPNPs showed potent wound-healing promotion effects in an MRSA-challenged full-thickness-wound mouse model. Novel chemical NO donors and Pluronic F127 co-assembled nanoparticles have recently been produced with improved NO loading and antimicrobial properties [110]; however, the potential efficacy of these formulations in vivo has yet to be determined. 

### 3.3. Phototherapy

**Figure 5:**
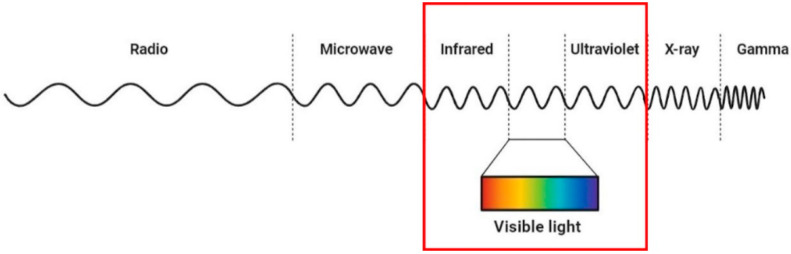
Electromagnetic spectrum. The region of the spectrum used for phototherapy is highlighted.

Phototherapy or “light therapy” is a medical treatment in which natural or artificial light is used to improve a health condition. In his work entitled *The history of phototherapy: something new under the sun?*, Roelandts, R. describes the hallmarks in the history of phototherapy [111]. One of the first reports of sunlight being used to treat infections (heliotherapy) is from 1400 BC, in which people with vitiligo were treated with some plant extracts and then exposed to the sun [112]. Since the 19th century, many advances have been made in the field. Heliotherapy was used for the treatment of peritoneal tuberculosis, rickets, and lupus vulgaris [113]. Over the years, it was discovered that part of the bactericidal effects could be attributed to the blue–violet region of the solar spectrum, and that oxygen was necessary for those effects [111]. The knowledge of the role of UV rays in the therapeutical effects of the light led to the development of artificial sources of light, which led to the initiation of phototherapy. The region of the electromagnetic spectrum emitted by the sun that is used for phototherapy treatments is depicted in Figure 5. It has been demonstrated that UV, visible light, and infrared rays are able to kill *S. aureus*. UV and violet–blue wavelengths produce photochemical effects, whereas infrared rays produce photothermal damage [114,115,116]. Depending on the wavelength, the dose, the duration, the frequency of the exposure, and the presence or absence of exogenous agents such as photosensitizers, phototherapy can lead to either the inactivation of microorganisms or their death. 

#### 3.3.1. Photodynamic Therapy

Photodynamic therapy (PDT) combines the administration of photosensitive compounds (photosensitizers, PSs) with the subsequent irradiation of the affected area with visible or near infrared light. PS compounds absorb energy from specific wavelengths and transfer it to molecular oxygen. Cytotoxicity is exerted by the generation of ROS by two mechanisms: electron transfer to produce superoxide radical anion O_2_^−^ or energy transfer, which produces the electrophilic singlet oxygen ^1^O_2_. The generated ROS causes photo-oxidative stress on organic molecules such as lipids and proteins that are part of the bacterial envelope, leading to bacterial death without acquiring new resistance. However, one of the disadvantages of using PDT involves the lack of selectivity for microorganisms [117]. Photosensitizers play a crucial role in PDT. Several publications summarize in depth the mechanisms of action and evaluate the advantages and disadvantages of using the different PSs, the wavelength employed, the reduction in bacterial load post-treatment and the possible combination with other antimicrobial strategies [118,119,120]. Pérez et al. recently published a summary of the main PSs, developed to eradicate *S. aureus* using PDT. This classification included the type and the dose of the PS, the source and the dose of the light employed, if it was an in vitro or an in vivo study and the main effects observed [121].

There are numerous studies that have evaluated the possible effects of PDT on the viability of MRSA in vitro; among these, we highlight some combinations of PSs and light that significantly reduced bacterial counts. The red light was the most widely used light source in therapy. When it was combined with protoporphyrins (620–780 nm; 12–200 J/cm^2^) and applied to *S. aureus* clinical isolates, it produced a 3–4.5 log10 reduction in the number of CFUs. When the PS used was methylene blue or toluidine blue (620–670 nm; 10–200 J/cm^2^), a 94% reduction in biofilm formation and a 3–6 log10 reduction in CFUs were observed. 5-ALA combined with red light (633 nm, 383 J/cm^2^) induced complete disruption of the biofilm [121]. Among the studies that have evaluated the efficacy of PDT in vivo, topical application of fulleropyrrolidine iodide salt on murine wounds infected with MRSA followed by green light exposure (525 nm; 50 mW/cm^2^) 30 min post the administration of the photosensitizer led to a 2 log10 reduction in bacterial burden compared with non-treated mice [122]. Another study evaluated the PDT on rat wounds infected with *S. aureus*, using indocyanine green as the PS exposed to a laser diode (810 nm; 300 mW/cm^2^, 30 s) 30 min after sensitization. PDT reduced the absolute colonization of the wounds and accelerated wound healing [123].

Blue light (BL; 400–470 nm) mediates bacterial killing by inducing the photoexcitation of endogenous porphyrins present in bacterial membranes, resulting in the production of ROS, which, in turn, induces DNA damage and macromolecule peroxidation. This therapy seems to be highly selective against bacteria due to the high amount of porphyrins present in bacterial membranes compared with mammalian cells. However, the disadvantage of using BL is the deficient tissue penetration. The main effects of BL are well summarized in the literature [124,125]. The antibacterial efficacy of BL against *S. aureus* has been demonstrated using wavelengths between 455 nm and 470 nm. In studies using planktonic MRSA, a greater than 5 log10 decrease in the number of CFUs was observed after exposures of 54 to 108 J/cm^2^ BL (400 nm) [126] and 133 J/cm^2^ BL (405 nm) [127]. Bacteria exposed to 28.5 J/cm^2^ BL (412 nm and 450 nm) showed a reduction of 72% (412 nm) and 81% (450 nm) in the number of CFUs, and in both cases, the addition of riboflavin improved the antimicrobial efficacy [128]. Moreover, in an attempt to optimize the parameters that can influence the efficacy of BL, Biener et al. demonstrated that two administrations of BL were more effective in inactivating MRSA than a single application with the equivalent exposure [129]. The efficacy of BL has also been evaluated in vivo. Mice infected with MRSA and treated with BL at 460 nm (120 J/cm^2^) daily for 2 weeks had reduced bacterial loads in the wounds, improved wound healing, and an increased survival rate [130].

BL can also be applied in the presence of an exogenous PS, and curcumin is one of the most commonly used. In a recent study, Akhtar et al. demonstrated the efficacy of employing curcumin as the PS and a diode laser (405 nm) against a biofilm of Vm-resistant *S. aureus* (VRSA) in vitro and in vivo. In this case, intracellular ROS accumulation led to bacterial death without generating human cell toxicity [131]. The combination of curcumin and BL was also effective against MRSA in a murine model of intradermal infection. Briefly, mice were intradermally inoculated with 10^8^ CFUs of *S. aureus*, given curcumin at the infection site, and left in the dark for 30 min. Then, mice were treated with LED light (450 nm, 10 min, 54 J/cm^2^). The authors found that this PDT reduced the bacterial load in the draining lymph nodes as well as the hyperplasia of these organs compared with the control groups, suggesting that this therapy can improve the clearance of bacterial infection [132].

Another PS evaluated in combination with BL was pyocyanin from *P. aeruginosa* [133]. Two different *S. aureus* strains were selected to evaluate the effects of pyocyanin BL therapy in vitro and in vivo: a staphyloxanthin-producing MRSA strain and the multidrug-resistant VISA strain AR0215. For in vitro assays, different BL (405 nm) exposures were combined with different concentrations of pyocyanin. When BL (216 J/cm^2^) was administered alone, for both strains (in a planktonic state), it resulted in less than a 1.5 log10 reduction in the number of CFUs, but in combination with the minimal pyocyanin dose used (6.25 µg/mL), a greater than 4 log10 decrease in CFUs was observed [133]. Pyocyanin potentiated the BL killing of both strains in a dose-dependent manner, reaching the maximum bacterial reduction (more than 5.5 log10 CFU) with 25 µg/mL of pyocyanin and 162 J/cm^2^. Moreover, all the concentrations of pyocyanin used improved BL’s ability to kill MRSA in biofilms, with 25 µg/mL of pyocyanin and 216 J/cm^2^ of BL (405 nm) resulting in more than a 4 log10 reduction in bacterial CFUs [133]. To evaluate the efficacy of this treatment in vivo, a murine model of skin abrasion and MRSA infection was used. Applying 25 µg/mL of pyocyanin with 216 J/cm^2^ of BL during the early onset of skin infection induced a 99% reduction in bacterial load in the infected wounds, without affecting normal skin [133].

Another strategy that has been evaluated in vitro and in vivo is the use of antibacterial photodynamic peptides, which are AMPs conjugated with a PS; in this case, chlorin e6 (AMPs-Ce6). The peptides were mixed with the bacterial culture (10^7^ CFU) for 1 h and then irradiated with an NIR laser (660 nm, 0.8 W/cm^2^) for 6 min. AMP2-Ce6 (4 μM) was able to kill *S. aureus*, and NIR markedly increased the bactericidal activities of all three AMPs. Co-incubation of AMP2-Ce6 with *S. aureus* for 2 days and subsequent IR irradiation significantly inhibited the formation of a biofilm. Finally, AMP2-Ce6 applied to *S. aureus*-infected skin wounds by a Gel/Col hydrogel demonstrated antibacterial activity and skin regeneration properties, using the same NIR irradiation (660 nm, 0.8 W/cm^2^, 6 min) for 10 consecutive days [134].

#### 3.3.2. UV Phototherapy

UV radiation (UVr) comprises a range of wavelengths between 100 and 400 nm and can be subdivided into UVC (100–280 nm), UVB (280–320 nm), and UVA (320–400 nm). UV effects can be produced by two different mechanisms. UVr can be absorbed by endogenous chromophores, leading these PSs to a differential excitation state. Subsequently, this energy is transferred to DNA in the presence of oxygen, causing oxidative damage to DNA, proteins, and lipids. A second mechanism involves the direct absorption of energy by the DNA, causing detrimental damage such as the formation of cyclobutene pyrimidine dimers (CPDs) and (6,4) photoproducts, which might cause defects in cell replication and lead to cell death [135]. UVA mainly causes oxidative stress, whereas UVB and UVC cause DNA photoproducts. The penetration of UVr varies according to the wavelength used, and it is lower for the shorter wavelengths.

UVC is known to be highly germicidal, and it seems to be the best at inactivating microorganisms. UVC is used to treat superficial infections or to decontaminate environmental surfaces. However, prolonged and repeated exposure to UVr can damage the host cells. Wavelengths between 250–270 nm are strongly absorbed by nucleic acids of microbial cells, causing DNA and RNA damage that alters cell replication, leading to death. Therefore, the challenge is to find a specific UVC wavelength that can produce antimicrobial effects without damaging host cells. Yin et al. summarized some in vitro studies employing UVC irradiation for the inactivation of bacteria, including *S. aureus*. Most of them were performed using 254 nm at a wide range of exposures from 5 to 15 mW/cm^2^; another work employed 265 nm of UVC light (1.93 mJ/cm^2^). In all cases, a 99–100% rate of bacterial inactivation has been reported [136]. 

Very few studies have focused on evaluating the effects of UVC irradiation using in vivo models. Some of them evaluated wavelengths between 270–280 nm, because it is known that tryptophan and tyrosine absorb this energy, which destroys the protein structure of microbes, with less DNA damage. Song et al. recently analyzed the effects of using a wavelength of 275 nm on different bacteria and fungi [137]. UVC-LED light inactivated a broad range of bacteria, including *S. aureus*, in a dose-dependent manner. Increasing the irradiation time from 5 s (7 mJ/cm^2^) to 60 s (90 mJ/cm^2^) resulted in an in vitro decrease in bacterial CFUs, reaching complete inactivation after 60 s. Moreover, it has been demonstrated that UVC-LED light reduced the wound-healing time in mice with skin and soft tissue infections induced by MRSA. Wounds were UVC-irradiated for 0, 10 (50 mJ/cm^2^), 20 (100 mJ/cm^2^), or 60 (300 mJ/cm^2^) seconds [137]. No significant differences were found during the first 4 days after UV irradiation. However, in the group of mice irradiated for 60 s, the scab fell off earlier than in the other groups. Moreover, the scab area was significantly reduced (34.66% unhealed), and the wounds disappeared on day 12. At the same time point, the non-irradiated group had a 48% unhealed wound [137]. The histopathological analyses revealed that no MRSA clumps were observed in the 60 s irradiation group. Moreover, neovascularization and fibroblasts were observed in the subcutaneous tissue. The load of MRSA in the wound correlated with the wound-healing process. Therefore, UVC-LED therapy inactivates the bacteria in the wound. Finally, the effects of a single 60 s exposure on keratinocytes were evaluated. Cyclobutane-pyrimidine-dimer-expressing (CPD+) cells were immediately detected, mainly in the stratum spinosum. Six hours later, these cells were localized in the upper stratum spinosum. Only a few CPD+ cells were detected 24 h later. When analyzing multiple 60 s exposures, no CPD+ cells were detected at 3 days post-irradiation, which serves as an indicator of the safety of the treatment [137]. 

#### 3.3.3. Photothermal Therapy

Photothermal Therapy (PTT) is a novel treatment in which photothermal agents (PTA) transform light energy into heat, producing membrane disruption, protein denaturalization, and bacterial destruction. Near infrared light (700–950 nm) is the most suitable light for PTT because of its excellent capacity to penetrate tissues with minimal damage to the host cells. PTAs, which are either nanomaterials themselves or small molecules loaded onto nanoparticles, play a crucial role in PTT’s effectiveness. As with PS and PDT, in PTT, the choice of PTA is essential for therapy success. Chen et al. summarized some of these PTAs and their applications in antibacterial PTT, including noble-metal nanomaterials, metal-sulfide/oxide nanomaterials, carbon-based nanomaterials, polymer-based nanomaterials, small-organic-molecule-based nanomaterials, and possible combination strategies [138]. One example of the use of PTT against *S. aureus* has been shown by Naskar et al. The authors employed a Au-ZnO-BP nanocomposite against MRSA, which combines three nanomaterials, Au, ZnO, and black phosphorous (BP) [139]. Considering the antibacterial and drug resistance properties of Au and ZnO, as well as the photothermal characteristic of Au and BP nanoparticles, the authors proposed a new nanoplatform using these nanoparticles (AZB) and NIR-irradiation (808 nm; 2.5 W, 5 min) to evaluate antibacterial effects against *S. aureus*, which was verified by its photothermal effect, bacterial growth curves, and SEM images. After irradiating the bacteria treated with AZB, the growth rate was decreased, and almost no bacterial growth was observed after 20 h. These bacteria showed notably disrupted and wrinkled membranes with morphological defects, as shown by SEM images.

### 3.4. Magnetic Nanoparticle Hyperthermia

**Figure 6:**
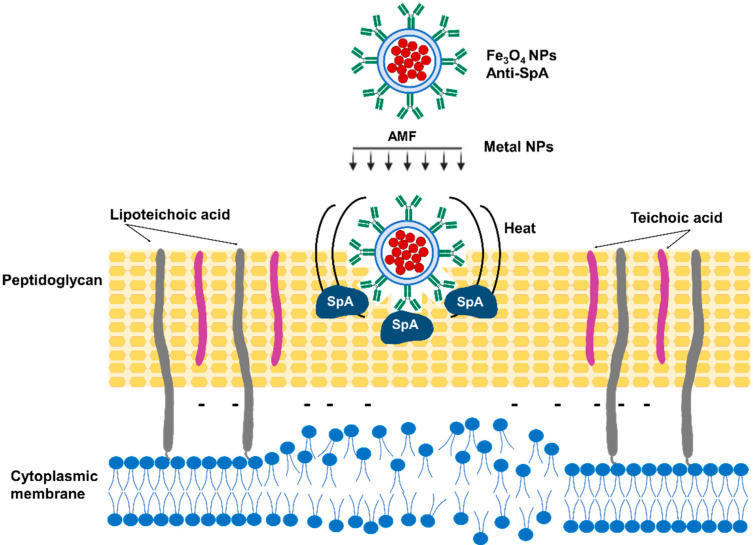
*S. aureus*-targeted magnetic nanoparticle hyperthermia. Magnetite (Fe_3_O_4_) coated with streptavidin and a biotinylated anti-protein A (SpA) antibody (anti-SA) binds to the *S. aureus* cell wall, and the alternating magnetic field (AMF) can induce thermal inactivation.

Kim Min-Ho et al. used targeted antimicrobial magnetic nanoparticle (MNP) hyperthermia to treat *S. aureus*-infected wounds [140]. The nanoparticles are able to absorb electromagnetic radiation and transform this energy into heat around the surface of the particle [141]. MNP hyperthermia has been successfully used for the treatment of glioblastoma, prostate carcinoma, and breast carcinoma [142,143,144]. The MNP dosing and the magnetic field applied are key factors that require careful regulation to prevent unwanted damage to healthy cells in the surrounding tissue. *S. aureus*-targeted MNPs were produced using magnetite (Fe_3_O_4_) coated with streptavidin and a biotinylated anti-staphylococcal protein A antibody (anti-SA). Conjugation of MNPs to anti-staphylococcal antibodies facilitates specific binding to the *S. aureus* surface and induces thermal inactivation [140] (Figure 6). The antimicrobial effect was evaluated in vitro using preformed biofilms treated with anti-SA-MNPs. An alternating magnetic field (AMF) of 31 kA/m for 3 min was then applied to treated and non-treated biofilms. Anti-SA-MNPs considerably improved *S. aureus* inactivation compared with either IgG-MNPs or non-targeted MNPs, with approximately 70% killing for anti-SA-MNPs and 30% for the controls. The results were confirmed with a live/dead viability assay based on the fluorescent staining of SYTO9 and PI uptake. The potential of anti-SA-MNP thermotherapy was then assessed using a mouse model of *S. aureus* cutaneous wound infection (1 × 10^7^ CFU/wound). Two days post-infection, anti-SA-MNPs were injected into the wound, and 2 h after that, an AMF (31 kA/m for 3 min, the same as used for the in vitro biofilm disruption) was applied. PBS-injected animals were used as controls. The bacterial burden before and immediately after AMF treatment was determined using whole-animal bioluminescence imaging, considering that the intensity of the *S. aureus* bioluminescent signal proportionally correlates with the bacterial burden in wounds [145,146]. Mice treated with anti-SA-MNPs or IgG-MNPs experienced similar increases in wound temperature, and neither physical harm nor unfavorable side effects were noticed after AMF treatment. Moreover, wound healing in the mice treated with anti-SA-MNPs was considerably improved compared with that observed in mice treated either with IgG-MNPs or receiving no therapy [140]. 

### 3.5. Mercaptophenylboronic Acid-Activated Gold Nanoparticles

Mercaptophenylboronic acid (MBA) has a mercapto group that can bind to AuNPs via an Au-S bond, and a boronic acid group that can bind covalently to the glycolipids on the bacterial cell wall [147,148]. MBA then may operate as a linker molecule between AuNPs and Gram-positive bacteria. Au-MBANPs produced by the NaBH_4_ reduction of HAuCl_4_ in the presence of MBA efficiently prevented multi-drug resistant *S. aureus* growth with an MIC of 6 μg/mL [149].

Bacterial permeability changes in the presence of the Au-MBANPs were evaluated using SYTO9/PI labelling. Treatment of *S. aureus* with Au-MBANPs increased bacterial permeability in a concentration-dependent manner. *S. aureus* was examined using SEM imaging after being exposed to different doses of Au-MBANPs. Imaging analysis indicated that the majority of the Au-MBANP-treated bacteria blend together with no discernible distinct cell envelope structure, indicating bacterial breakdown [149]. The Au-MBANPs exhibit no in vivo toxicity at an exceptionally high concentration (200 times the MIC), which further supports their outstanding biosafety as nano-antimicrobials.

Wang et al. fabricated fibrous matrices of poly(ε-caprolactone) and gelatin (PG) with Au-4MBANPs (PGANPs). The antibacterial activity of the PGANPs was evaluated using *S. aureus*-infected full-thickness skin-wound models in rats. The wound-healing rates in the PG-treated and PGANP-treated groups were significantly higher than that in the control group (gauze without treatment) at the same time points due to the improved wound healing induced by PG. The PGANP-treated group on day 10 post-infection presented a significantly reduction in the wound size compared with the other groups [149]. The fibrous PG and PGANP matrices may accelerate the healing process, as evidenced by the early emergence of elongated fibroblasts on day 7, as opposed to day 14 for the control group. The immune inflammatory infiltrates disappeared in the PGANP group by day 14, whereas they were still present in the wounds of the PG-treated and control groups at the same time point. Additionally, the skin of the PGANP-treated group presented organized collagen fibers as well as an increased amount of hair follicles and sebaceous glands, whereas the PG-treated group had fewer of these structures, and none of these were present in the control group [149]. These findings indicate that PGANPs not only promote bacterial elimination but also contribute to the resolution phase of the wound infection and tissue repair. 

### 3.6. Dialdehyde Nanocrystalline Cellulose

Nanocrystalline cellulose (NCC) is a highly biocompatible nanomaterial that is easily modified with high chemical activity and has a large surface area [150,151,152,153]. NCC has been used to produce dialdehyde nanocrystalline cellulose (DNC) by the sodium periodate-mediated oxidation of hydroxyl groups on C2 and C3 to aldehyde groups [154]. The antibacterial properties of DNC and NCC against MRSA were evaluated using the bacteria growth-curve method and the colony-counting method. DNC completely inhibited *S. aureus* growth and killed bacteria after 24 h of incubation (initial concentration: 10^7^ CFU/mL); whereas NCC slightly reduced growth compared with the untreated control, with no effect on CFU counts. The smaller particle size, the shift in shape from rod to needle, and the higher aldehyde group concentration in DNC compared to NCC may have improved the interaction with bacteria, which could account for the increased antibacterial activity [129,155]. 

The mechanism by which DNC presents an antibacterial effect has been studied by observing the morphology of *S. aureus* after cultivation in the presence of this nanomaterial. The *S. aureus* diameter was significantly larger in DNC-treated cultures compared with the control, being 0.85 ± 0.02 μm and 0.43 ± 0.03 μm respectively. In addition, SEM images showed destruction of the cell membrane, which produced an outflow of cellular contents and, ultimately, the inactivation of the pathogen [156,157,158]. DNC antibacterial activity was also evaluated in a mouse model of skin colonization, with healthy and injured skin exposed to 10^7^ CFUs of MRSA. Mice were then treated with 2.5 mg of DNC or with PBS (control group), and the number of CFUs in the skin was determined. DNC treatment significantly reduced *S. aureus* skin colonization by 2 log10 compared with the control, not only in injured, but also in uninjured skin. DNC has an outstanding bactericidal activity against MRSA and, considering that the development of resistance is unlikely, DNC constitutes a promising treatment for staphylococcal SSTIs.

### 3.7. Bioactive Glass Nanoparticles

Zhang et al. developed mannose-modified bioactive glass nanoparticles decorated with silver nanoparticles (Man-BGNs/Ag) to treat *S. aureus* skin infections [159]. To produce the Man-BGNs/Ag particles, mannose was first conjugated to the BGNs and then Ag^+^ ions were reduced with glucose to form Ag nanoparticles on Man-BGNs [160]. Due to the release of Ag^+^ and the induction of ROS [161], the nanoparticles were expected to kill *S. aureus*. The bactericidal effect was verified in *S. aureus* cultures by SYTO 9/PI labelling and CFU counts. 

Macrophages are key phagocytic cells involved in the induction of inflammatory responses, neutrophil recruitment, and bacterial clearance during *S. aureus* skin infections [162,163]. Macrophage-mediated inflammation is also critical to orchestrate the transition to anti-inflammatory M2-like phenotypes that modulate tissue repair and wound healing [164]. *S. aureus* can escape phagocytic killing and survive intracellularly in macrophages, which compromises its role in infection and might promote bacterial spread [165]. Nanoparticle mannose coating is a strategy that has been successfully used to target macrophages due to their abundant mannose receptors [166,167,168,169]. Mannose coating in Man-BGNs/Ag could increase their internalization in macrophages and, therefore, intracellular bacterial clearance during infection. Infected macrophages were treated with Man-BGNs, Man-BGNs/Ag, or vancomycin, and the survival of intracellular *S. aureus* was determined. Neither Man-BGNs nor vancomycin efficiently eliminated intracellular *S. aureus*, as determined by SYTO9/PI staining. However, Man-BGNs/Ag inactivated most of the intracellular bacteria. Apart from the effect of Man-BGNs/Ag on the bacteria, the NPs induced ROS production by macrophages, further improving their intracellular antibacterial efficiency [159]. The potential efficacy of the Man-BGNs/Ag in vivo treatment was evaluated in a murine skin abscess infection model. On day 7 post-infection, Man-BGNs/Ag treatment significantly reduced bacterial burden in skin abscesses compared with BGN-treated, Man-BGN-treated and untreated mice. As expected, a reduction in the bacterial burden resulted in an increased number of M2 macrophages and, therefore, accelerated wound healing [159].

### 3.8. Antisense Oligonucleotides Targeting Essential Genes

Antisense peptide nucleic acids (PNAs) are synthetic DNA analogs of antisense nucleic acids. PNAs targeting the mRNA for *ftsZ*, an essential gene for bacterial cell division, were used to inhibit MRSA growth in vitro [170,171]. In order to improve stability and cell penetration, PNAs were conjugated with a bacterial-cell-penetrating peptide (P-PNAs) [170] or a non-cytotoxic tetrahedral DNA nanostructure (TDN-PNAs) [171]. The expression of *ftsZ* was successfully inhibited by P-PNAs and TDN-PNAs in a concentration-dependent manner, as demonstrated by transcriptional analysis using RT-PCR or qPCR, respectively [170,171]. MRSA growth in vitro was proportionally inhibited as *ftsZ* expression was reduced. The TDN system efficiently delivered PNAs into drug-resistant bacterial cells and induced a 60% inhibition in growth at a concentration of 750 nM, whereas P-PNAs required concentrations higher than 30 µM to reduce growth to 50% [170,171]. Antisense oligonucleotides targeting *ftsZ* were also evaluated in combination with silver nanoparticles using a DNA six-helix bundle nanostructure, and a synergistic antimicrobial effect due to the inhibition of *ftsZ* expression and Ag^+^ cell envelope destruction was observed [172]. 

## 4. Conclusions

Nanotechnologies have entirely changed the field of drug delivery by offering countless combinations and possibilities for drug stabilization and targeted delivery for both parenteral and local administration. In this review, we have thoroughly described the various platforms currently being investigated for novel treatments against *S. aureus* skin and soft tissue infections. The preclinical data obtained so far suggest that these treatments could enhance bacterial eradication, lessen side effects, and, in some cases, even reduce the frequency of drug-resistance development. Table 1 lists the specific strategies to overcome the resistance and/or bioavailability issues encountered with certain antibiotics. The strategies under development include antibiotic-based and non-antibiotic-based approaches. Considering that SSTIs are a huge burden on public health and a reservoir for multidrug-resistant bacteria, particularly MRSA, local treatments that do not rely on antibiotics represent an interesting novel approach. The development of resistance using non-antibiotic-based strategies is unlikely since most of them have multiple effects on *S. aureus* that result in bacterial death, which is a significant improvement over antibiotics that typically have one specific target. Additionally, several of the strategies reviewed here not only contribute to bacterial eradication but also promote wound healing. Future research using in vivo models will provide the necessary knowledge to determine whether some of these approaches can be transferred to clinical studies.

## Figures and Tables

**Table 1:** Specific strategies to overcome antibiotic resistance and/or bioavailability.

Antibiotic	Mechanism of Action	Therapeutical Problem	Alternative Strategies	Ref.
**β** **-Lactam (penicillins, cephalosporins, and carbapenems)**	Interference with the synthesis of peptidoglycan	Antibiotic resistance: 1. β- Lactamases, *blaZ*	(1a) Combination with metal nanoparticles. (1b) Chemical enzyme inhibitors. (1c) Inhibition of *blaZ* expresion.	(1a) [11,12] (1b) [14] (1c) [13]
		2. *mecA* product PBP2a reduced affinity for PBP	(2a) Antisense technology to block PDP2a expression. (2b) Combination with ZA-S. (2c) Non-antibiotic-based treatments.	(2a) [16,17] (2b) [18] (2c) See Section 2.
**Vancomycin (Vm)**	Blocking of peptidoglycan crosslink formation through binding to (D-Ala-D-Ala)	1. Elevated toxicity and stability problems	(1a) Enhance antimicrobial activity with lipid-based nanoformulations. Vm-loaded polymersomes and OLA-LPHNVs1-Vm nanovesicles.	(1a) [50,51]
			(1b) Local treatments to enhance tissue penetration and skin accumulation of ATB. Vm-loaded nanobubbles and US and Vm-loaded chitosan nanoparticles coated with lecithin (CLNPs).	(1b) [54,55]
		2. Antibiotic resistance.	2. Non-antibiotic-based treatments Blue-light-mediated VISA and VRSA killing or in combination with PS.	2. [131,132,133]
**Quinolones**	Inhibition of bacterial replication	High toxicity and low solubility	Nanoformulations that increase biodisponibility and reduce antibiotic dosis: (1a) Solid dispersions (SD); AgNPs-PEG-Cip (1b) Lipidbased formulations; Pluronic F127/Cremophor EL-Nfx and niosomal vesicles (MNV) as Mox nanocarriers. (1c) Complex formations with cyclodextrins; CD-Nfx, CD-Mox	(1a) [25] (1b) [32,33] (1c) [30,31]
**Macrolides**	Inhibition of protein synthesis *50S inhibition*	High toxicity and solubility problems	1. Nanoformulations that increase bioavailability and reduce antibiotic dosage: (1a) Solid dispersions (SD); AZO-Em. (1b) Complex formations with modified cyclodextrins; CD-MAN-Em, CD-GLU-Em.	(1a) [23] (1b) [43]
			2. Local treatments that enhance tissue penetration and skin accumulation of ATB; Azt - Microemulsions (MEs).	2. [59]
**Tetracyclines**	Inhibition of protein synthesis *30S inhibition*	High toxicity and solubility problems	1. Local treatments that enhance tissue penetration and skin accumulation of ATB. Tig-loaded chitosan nanoparticles coated with lecithin (CLNPs).	1. [55]
